# A Response Assessment Platform for Development and Validation of Imaging Biomarkers in Oncology

**DOI:** 10.18383/j.tom.2016.00223

**Published:** 2016-12

**Authors:** Hao Yang, Lawrence H. Schwartz, Binsheng Zhao

**Affiliations:** Department of Radiology, Columbia University Medical Center, New York, New York

**Keywords:** imaging platform, WEASIS, quantitative imaging biomarkers, tumor response assessment, volumetry

## Abstract

Quantitative imaging biomarkers are increasingly used in both oncology clinical trials and clinical practice aid evaluation of tumor response to novel therapies. To obtain these biomarkers, and to ensure smooth clinical adoption once they have been validated, it is critical to develop reliable computer-aided methods and a workflow-efficient imaging platform for integration in research and clinical settings. Here, we present a volumetric response assessment system developed based on an open-source image-viewing platform (WEASIS). Our response assessment system is designed using the Model–View–Controller concept, and it offers standard image-viewing and -manipulation functions, efficient tumor segmentation and quantification algorithms, and a reliable database containing tumor segmentation and measurement results. This prototype system is currently used in our research laboratory to foster the development and validation of new quantitative imaging biomarkers including the volumetric computed tomography technique as a more accurate and early assessment method of solid tumor response to targeted therapy and immunotherapy.

## Introduction

The past decade has seen successful identification of oncogenic targets. For example, the ability to detect epidermal growth factor receptor mutations and the anaplastic lymphoma kinase rearrangements has increased therapeutic options for patients with lung cancer. However, the development of a tissue biomarker that can predict sensitivity to targeted therapy has relied on the conventional method of Response Evaluation Criteria In Solid Tumors (RECIST) (1, 2). RECIST uses unidimensional measurement (ie, maximal tumor in-plane diameter) to quantify tumor change during the course of therapy, which may limit the ability to distinguish drug-sensitive and -resistant tumors. This is because the unidimensional method cannot fully characterize tumor growth dynamics. In contrast, volumetric tumor measurement can capture change in total tumor burden (computed tomography [CT] volumetric technique) and has shown promise of being a more sensitive and early imaging biomarker for response assessment to (gefitinib) targeted therapy in non-small cell lung cancer (3). Besides the volumetric technique, there are many other quantitative and semiquantitative imaging biomarkers, particularly recent quantitative image features of Radiomics, that can be derived from positron emission tomography and magnetic resonance imaging and require clinical validation.

Realizing the urgent demand for improved response assessment methodologies in oncology clinical trials, the National Cancer Institute initiated a program in 2008, called Quantitative Imaging for Evaluation of Responses to Cancer Therapies, aiming to promote research of quantitative imaging methods pertinent to tumor response to therapies in clinical trial settings, with the ultimate goal of facilitating clinical decision-making (4). To date, 25 universities/cancer centers/hospitals (28 multidisciplinary teams) have been supported though the National Institutes of Health U01 grant mechanism and actively participated in the Quantitative Imaging Network. Our team, from Columbia University, is one of the Quantitative Imaging Network members and has been developing a new quantitative volume- and density-based response assessment method for solid tumors. To promote quantitative imaging biomarker development (ie, tumor volume and necrosis fraction), we have been developing segmentation algorithms for solid tumors (eg, tumors in lung, liver, and lymph node) and for necrosis on CT images (5–8). In addition, we have developed a prototype imaging platform that integrates our customized volumetric segmentation and quantification methods and allows efficient validation of the volumetric technique as a better method to assess tumor response to targeted therapy and immunotherapy.

Our response assessment system is based on an open-source image-viewing platform, WEASIS (9). Although there exist numerous open-source software platforms (10), we chose the WEASIS imaging platform as the foundation to build our overall platform. This is mainly because WEASIS offers several basic image-viewing and-manipulation functions that are provided by clinical Picture Archiving and Communication System (PACS); it allows plugins of customized programs written in C/C++ to extend the platform's capacity; and (although not necessary) the programming source codes of WEASIS are available. In the Methods of this report, we will explain in detail how we designed and implemented our response assessment system.

## Methods

### System Framework

**Figure 1.**
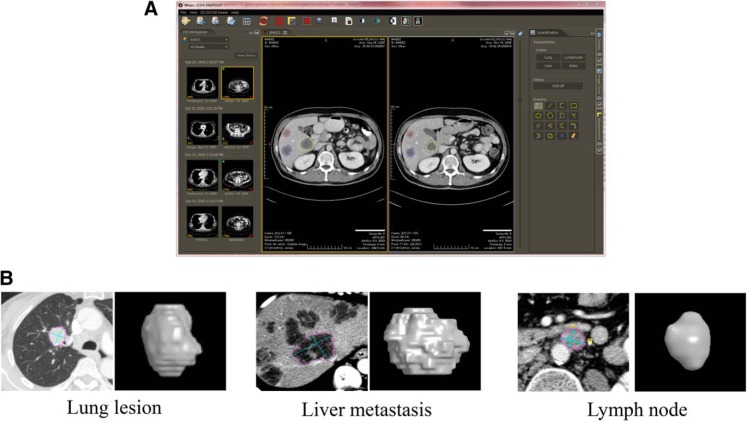
WEASIS-based response assessment system with integrated tumor segmentation and quantification algorithms (A). Lung, liver, and lymph node segmentation algorithms integrated into the WEASIS response assessment system (B).

Figure 1A shows the user interface of our WEASIS-based imaging platform for tumor response assessment. The user interface consists of a horizontal tool panel at the top for image viewing and manipulation (eg, image layout, zoom in/out, window/level (W/L) presetting, and maximum intensity projection) and 3 vertical panels for loading and navigating a patient's image (image series at each visit date are listed by small image icons) (Left), image display (Middle), and lesion quantification (eg, using the integrated segmentation and editing tools) (Right). For example, in the layout of 1 × 1, the center panel is split into 2 windows, one displays images from the baseline scan and the other displays images at a follow-up scan. In each window, 3 target liver lesions are selected and their contours are delineated using our segmentation algorithms and superimposed on the original images. Figure 1B shows examples of segmented lesions (and their 3-dimensional visualization) in the lung, liver, and lymph node. The uni- and bi-dimensional lines automatically extracted from the segmented lesion contours can also be calculated and displayed.

**Figure 2.**
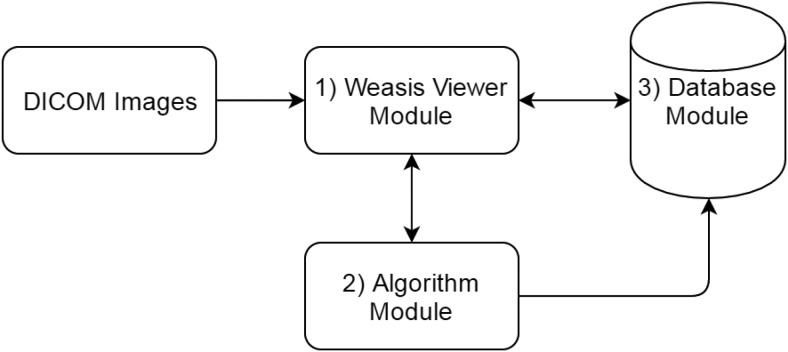
System framework diagram.

Our response assessment platform consists of the following 3 major components (Figure 2): the WEASIS viewer module to open, display, and manipulate radiological images; the algorithm module to integrate custom tumor segmentation and quantification algorithms; and the database module to store and manage lesion segmentation and measurement results. In the following sections, each of these modules and their interaction will be explained in detail.

#### The WEASIS Viewer Module.

WEASIS is a multipurpose Digital Imaging and Communications in Medicine (DICOM) viewer with a highly modular architecture. It facilitates uploading/storing of patient's DICOM images locally and/or from/to a database and provides standard image-viewing and-manipulating functions such as zoom in/out, pan, W/L, multiplanar reconstruction, and maximum intensity projection. It also provides W/L presetting for multiple body sites (eg, lung, abdomen, or mediastinum), custom color look-up tables, and multiple displaying window layouts (eg, 1 × 1, 1 × 2, 2 × 1, 2 × 2).

#### The Algorithm Module.

The algorithm module handles the integration of custom, advanced segmentation algorithms (eg, lung lesion, liver lesion, and lymph node segmentation algorithms and a set of lesion contour-editing tools) into the imaging platform. All algorithms are written in C++ and packaged into dynamic link libraries. Because WEASIS is programmed in Java, we use the Java Native Interface to call these algorithms/functions in the libraries.

Both lesion-segmentation and contour-editing functions have the standard input and output parameters. The input parameters include pointer to image volume data; image dimensions along x, y, and z directions; and the image resolution (pixel spacing and section thickness). The output parameters include a pointer to the image volume data containing the segmented lesions, the image dimension, and the resolution information. The lesion volume data are binary images in which the background pixels (2-dimensional)/voxels (3-dimensional) are 0 and lesion pixels are 1.

#### The Database Module.

**Figure 3.**
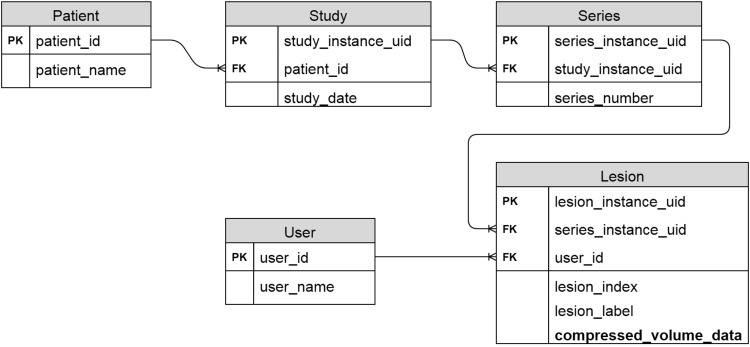
Relational database diagram (PK: primary key; FK: foreign key).

Patients' radiological images are stored in a standard structure, starting from the top level of study down to the levels of series and then image. One patient can have several imaging studies (ie, different modalities, clinical visits, or scan time points); each study can have multiple image series (eg, CT chest series, abdomen series); and each series can have a number of sectional images of an organ(s). Each of the 3 elements (study, series, and image) in this tree structure has a global unique identifier that can be read from the image DICOM header, which allows us to design a relational database to organize patient data. The diagram of our relational database is presented in Figure 3. In the database, there are 5 tables, namely, patient, study, series, user, and lesion, that are linked by the global unique identifiers. To reduce the space required for data storage, the DEFLATE compression algorithm is used to compress lesion volume data.

Our response assessment platform can support multiple users and allow >1 user to delineate the same lesion simultaneously and save their contours separately. This feature is convenient to conduct multireader studies when evaluating a quantitative method's accuracy and robustness. As shown in Figure 3, a user table is added to satisfy this requirement.

MySQL is used as the relational database management system, as it is secure, easy to use, scalable, and extremely powerful. We deploy the MySQL on a Red Hat Enterprise Linux (RHEL) server for reliability and efficiency.

### Software Design

**Figure 4.**
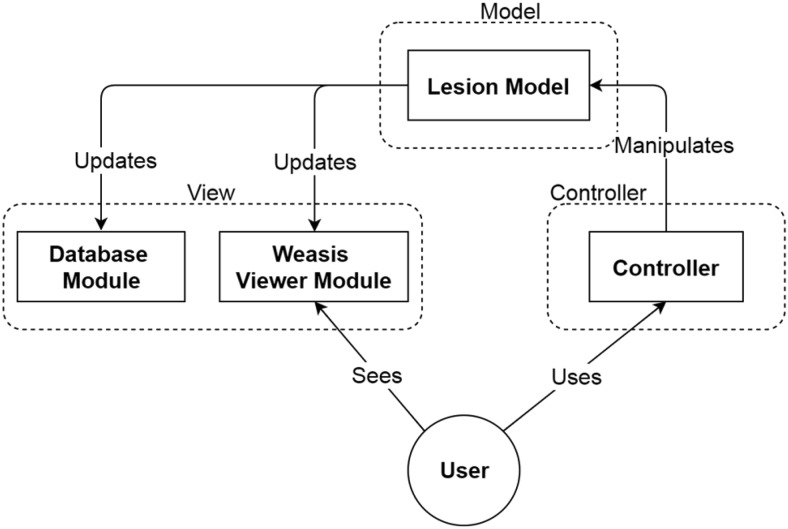
Schema of the Model–View–Controller (MVC) software design pattern.

The software design of our system follows the Model–View–Controller (MVC) pattern that separates our system into the following 3 interconnected parts: model, view, and controller (Figure 4).

As shown in Figure 4, the lesion model represents the MVC model concept in our system. It has a tree data structure with the following 4 layers from top to bottom: patient, study, series, and lesion. The 4 layers of the tree structure correspond to the 4 tables in the database module. Such data structure facilitates data storage and retrieval in the database module.

The lesion model has several essential functions. It can produce lesion boundary points by applying edge detection on the lesion volume data and connecting these boundary points to form a lesion contour. The WEASIS Viewer Module can display the generated lesion contour using a polygon graph. Another function of the lesion model is to compress the lesion volume data before saving the lesion contour result to the lesion table in the database and decompress lesion volume data when retrieving the tumor contour result from the lesion table in the database.

The controller accepts the input from the user, for example, a user clicking a lung lesion segmentation button using the computer mouse, and passing the request to the lesion model. The lesion model will then respond to the request by calling the corresponding dynamic link library (ie, the lung lesion segmentation algorithm) to perform the task.

The database module and the WEASIS viewer module can be considered as the view of the MVC concept. The MVC design allows separation between the model and the view in our system and makes our plugin modular and extensible. For example, the design of the graphical user interface and the database can be changed without changing the lesion model.

In such an MVC software design, both the WEASIS viewer module and the database module are observers of the lesion model. Thus, once the lesion model is changed, it will immediately trigger the database module and the WEASIS viewer module to update their statuses. This working mechanism can make programming easier. For example, when a user edits a segmented lesion, the controller accepts the computer mouse's operations and sends editing commands to the lesion model. Following the editing commands, the lesion model modifies the volume data of the segmented lesion. In response to the modification of the lesion model, the database module stores the updated lesion volume data to the database, and the WEASIS Viewer Module updates the lesion contour correspondingly.

### Response Assessment Workflow

**Figure 5.**
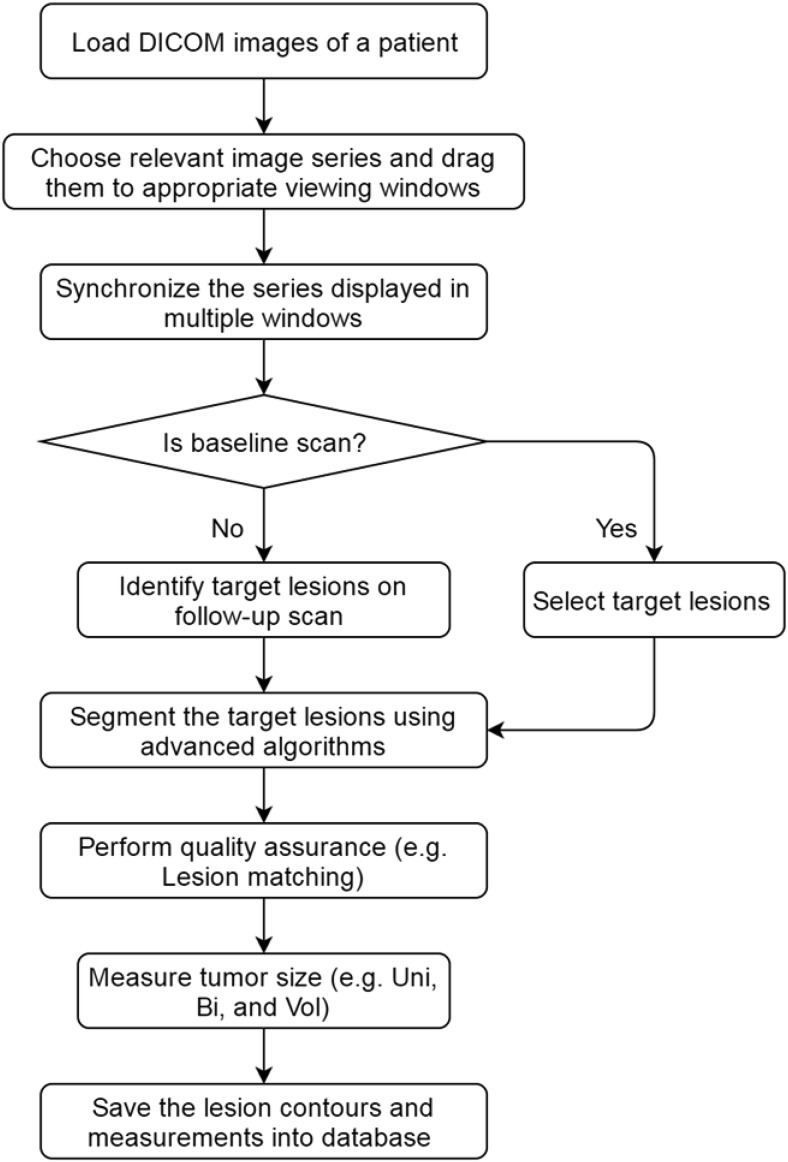
Workflow of response assessment.

The response assessment workflow is shown in Figure 5. When using this software to access therapy response, a user will first load all DICOM images of a patient from a disk or a PACS server, and choose by dragging proper image series to viewing windows. The viewing windows can be laid out as 1 × 1, 2 × 2, 4 × 4, etc. To efficiently review baseline and follow-up images, we can synchronize the image series at different scan time points. Further, the user will identify target lesions in the image set on the baseline images and segment the identified target lesions with the lesion segmentation algorithms. If any segmented results are suboptimal, the user can use the editing tools to correct them. Target lesions on each follow-up scan time point will then be identified, segmented, and measured. To ensure the correct determination of the target lesions at baseline and follow-up scans, we developed an automatic quality assurance algorithm that can help match the target lesions between different scan time points by using the lesion's relative position relationship in the patient coordinate system (11). After the quality assurance checking, the system will automatically calculate lesion unidimensionality, bidimensionality, and volume based on the final segmentation and save the segmentation and measurement results to the database.

## Results

We have developed a prototype imaging platform to support the development and validation of quantitative imaging biomarkers for improved assessment of (solid) tumor response to therapies, particularly novel targeted therapy and immunotherapy. This system is based on the open-source imaging platform WEASIS that provides basic DICOM image-viewing and-manipulation functions as seen in commercial clinical PACS. The WEASIS imaging platform allows customized functions/software (eg, tumor segmentation) to be integrated into the system through its plugin architecture, which is flexible for system developers and can be widely extended to different clinical applications.

Our system is currently under evaluation and used in a number of clinical trial studies including 2 large phase II/III clinical trials to investigate if CT volumetry- and density-based techniques can assess tumor response to targeted therapies more accurately and quickly than conventional RECIST. The first trial is a retrospective sarcoma clinical study. To date, we have completed the following sarcoma study: “A phase II trial of R1507, a recombinant human monoclonal antibody to the insulin-like growth factor-1 receptor for the treatment of patients with recurrent or refractory Ewing's sarcoma.” In this study, using our response assessment system, we delineated and measured target lesions in 101 patients with sarcoma (303 scan time points). We explored the prediction power of the response assessment metrics of volume and unidimensional and bidimensional measurements in overall survival and found that the volumetric technique is superior to RECIST or World Health Organization (WHO) (a bidimensional measurement method) in identifying tumor response (12). The second trial (total patients, 207; scan time points, 681), “A phase II/III randomized study of Sorafenib plus Doxorubicin versus sorafenib in patients with advanced hepatocellular carcinoma (HCC),” is currently under evaluation.

## Discussion

Our response assessment system has shown value in its ability to efficiently obtain/measure tumor size, particularly tumor volume, at serial scan time points in clinical trial settings to help monitor change in total tumor burden—a potential better imaging biomarker of response. We plan to further improve our system's workflow efficiency by (1) providing prehanging imaging protocols for image interpretation; (2) automating identification of target lesions on follow-up scans (this can be done through the already selected target lesions on the baseline scan and image registration between baseline and follow-up scan images); (3) automating identification of target lesions on baseline scan and new lesions on follow-up scans; (4) strengthening the system's quality assurance capability; and (5) optimizing reporting output format.

We will integrate our custom-developed radiomic features into the system so that it can be used to explore tumor imaging phenotypes for therapy response prediction and patient stratification for future clinical trials. We also plan to extend our response assessment system to study both positron emission tomography- and magnetic resonance imaging-derived imaging biomarkers.
